# Neuroprotective Effects of Heat-Killed *Levilactobacillus brevis* KU15152 on H_2_O_2_-Induced Oxidative Stress

**DOI:** 10.4014/jmb.2304.04045

**Published:** 2023-05-29

**Authors:** Hyun-Ji Bock, Na-Kyoung Lee, Hyun-Dong Paik

**Affiliations:** Department of Food Science and Biotechnology of Animal Resources, Konkuk University, Seoul 05029, Republic of Korea

**Keywords:** *Levilactobacillus brevis*, gut–brain axis, oxidative stress, heat-killed probiotics, neuroprotective effect

## Abstract

This study proposed to demonstrate the neuroprotective effects of heat-killed *Levilactobacillus brevis* KU15152. Heat-killed *L. brevis* KU15152 showed antioxidant activity similar to that of *Lacticaseibacillus rhamnosus* GG, in terms of radical scavenging activity. To evaluate the neuroprotective effects, conditioned medium (CM) obtained by incubating heat-killed bacteria in intestinal cells (HT-29) was used through gut-brain axis. CM from *L. brevis* KU15152 protected neuroblastoma cells (SH-SY5Y) against H_2_O_2_-induced oxidative stress. Pretreatment with CM significantly alleviated the morphological changes induced by H_2_O_2_. Heat-killed *L. brevis* KU15152 showed an increased brain-derived neurotrophic factor (*BDNF*) expression in HT-29 cells. *L. brevis* KU15152–CM remarkably downregulated the *Bax/Bcl-2* ratio, while upregulating the expression of *BDNF* and tyrosine hydroxylase (*TH*) in SH-SY5Y cells. Furthermore, *L. brevis* KU15152–CM reduced caspase-3 activity following H_2_O_2_ treatment. In conclusion, *L. brevis* KU15152 can be potentially used as food materials to avoid neurodegenerative diseases.

## Introduction

Oxidative stress is triggered when the balance between reactive oxygen species (ROS) generation and the capability to detoxify active intermediates is disrupted [1]. Excessive ROS generation have been associated with diverse neurodegenerative diseases involving Parkinson’s disease (PD), Huntington disease, and Alzheimer’s disease [2]. ROS, including H_2_O_2_, hydroxyl radicals, and superoxides, induce protein aggregation, mitochondrial dysfunction, and DNA damage, ultimately result in probable cell death [3, 4]. H_2_O_2_ is regarded as a representative ROS contributor that serves as a precursor of extremely reactive hydroxyl radicals [5]. The accumulation of H_2_O_2_ in neuronal cells spontaneously leads to apoptosis and neuronal damage, causing changes in function and morphology and resulting in progressive degradation of memory and cognition. Additionally, brain-derived neurotrophic factor (BDNF) and tyrosine hydroxylase (*TH*) are the critical neuronal biomarker in the neuroprotection. BDNF modulates the survival, differentiation, and proliferation of dopaminergic neurons and TH plays an important role in the dopamine biosynthesis pathway [6, 7].

Interactive signaling between the brain and the gut microbiota is critical for homeostasis [8]. The gut–brain axis (GBA) is an interactive neuroendocrine system comprising immunological factors, direct neurological connections, and endocrine signals [9]. GBA suggests the novel remedies to increase the disorders related to cognitive function and mental health [8]. Probiotics can be used as a potential therapeutic tool through the interlocking of biochemical and hormonal pathways related to GBA [9].

Probiotics are alive microorganisms that provide advantage to the host when treated in appropriate quantities. Probiotics have been used as potential nutritious ingredients for the treatment of diseases, such as colon inflammation and neuronal disorders [10]. Probiotics such as lactobacilli can produce bioactive molecules and antioxidants [11]. Consequently, they have the ability to decrease excessive amounts of free radicals and attenuate several disorders related to oxidative stress, such as PD [12, 13]. In addition, probiotics can generate various neuroactive compounds including gamma-aminobutyric acid, dopamine, acetylcholine, and serotonin [14]. Several PD animal models have suggested that probiotics have neuroprotective effects by decreasing dopaminergic neuronal degeneration [15, 16].

Safety issues regarding the use of live microorganisms remain [17]. In particular, probiotic bacteria can cause infections or autoinflammatory diseases when administered as living organisms [18]. To prevent these risks, many studies have employed heat-killed bacteria, purified components, and their fractions. Among these forms of bacteria, heat-inactivated lactic acid bacteria (LAB) have called as parabiotics and reported on favorable effects, along with antioxidant, anticancer, and anti-inflammatory effects [19]. In addition, the health function of heat-killed bacteria depends on probiotic strain.

*Levilactobacillus brevis* KU15152 was isolated from kimchi. In our previous study, we reported that *L. brevis* KU15152 can be utilized as a probiotic strain with anti-inflammatory effects [20]. However, the protective effects of *L. brevis* KU15152 in neuronal cells against oxidative stress have not yet been described. In terms of neuroprotection related to apoptotic pathway, neuronal cells may show the following indication: increase of cell viability, alleviation of cell morphology, upregulation of *BDNF* and *TH*, downregulation of the ratio of *Bax/Bcl-2* and inactivation of caspase-3 [21, 22]. Hence, this study was examined the neuroprotective effects of heat-killed *L. brevis* KU15152 in H_2_O_2_-induced SH-SY5Y cells for functional ingredients.

## Materials and Methods

### Bacterial Strains and Sample Preparations

*Levilactobacillus brevis* KU15152, KU15159, and KU15176 were isolated from fermented foods. *Lacticaseibacillus rhamnosus* GG (LGG) was taken from the Korean Collection for Type Cultures (KCTC, Korea) and treated as reference. LAB strains were cultured in MRS broth (Difco Laboratories, USA) at 37°C for 18 h. The strains in MRS broth were mixed with 20% (v/v) glycerol as 1:1 ratio and stored at –80°C until use. Each cultured strain was centrifuged at 14,240 ×*g* at 4°C for 5 min. The strains were rinsed twice and suspended in PBS (Hyclone, USA). Live bacterial cells were killed at 80°C for 30 min.

### Culture Conditions

HT-29 (human colon adenocarcinoma; KCLB 30038) and SH-SY5Y (human neuroblastoma; ATCC CRL-2266) cells were cultured in RPMI 1640 (Hyclone) and DMEM (Hyclone). Each medium included fetal bovine serum (FBS; 10% (v/v); Hyclone) and penicillin–streptomycin (1% (v/v); Hyclone). Each cell was incubated at 37°C in a humidified atmosphere containing 5% CO_2_.

### Antioxidant Activity of Heat-Killed LAB Strains

DPPH (2,2-diphenyl-1-picrylhydrazyl) radical scavenging activity was measured according to the method described by Song *et al*. [23], with some modifications. Five hundred microliters of 0.1 mM DPPH solution were dissolved in ethyl alcohol and mixed with the same amount of a heat-killed LAB suspension (1 × 10^9^ CFU/ml). After incubation at 25°C for 30 min, the mixture was centrifuged at 14,240 ×*g* for 1 min. The absorbance of the supernatant was measured at 517 nm, and the radical scavenging activity was determined using Eq. (1).

$${\text{Radical scavenging activity (\%) = (1-A}}_{\text{sample}}{\text{/A}}_{\text{control}}\text{)×100}$$ (1)

where A_control_ and A_sample_ represent the absorbance of the control treated with PBS and the sample treated with heat-killed LAB, respectively.

ABTS (2,2´-azino-bis-(3-ethylbenzothiazoline-6-sulfonic acid) diammonium salt) radical scavenging activity was determined as per the method depicted by Jang *et al*. [24], with some modifications. Firstly, 14 mM ABTS and 5 mM potassium persulfate were dissolved in distilled water and mixed at a 1:1 ratio. ABTS solution was incubated at 25°C for 16–18 h in the dark. The reacted ABTS solution was weakened using distilled water up to the final absorbance reached 0.7 ± 0.5 at 734 nm. Eight hundred microliters of diluted ABTS solution were blended with 200 μl of heat-killed LAB suspension (1 × 10^9^ CFU/ml), then incubated at 25°C for 15 min. Following centrifugation at 14,240 ×*g* for 1 min, the absorbance of the supernatant was evaluated at 734 nm. The ABTS radical scavenging activity was calculated using Eq. (1).

### Manufacturing of Conditioned Medium (CM) from HT-29 Cells

CM was prepared followed the method of Cheon *et al*. [25]. HT-29 cells were seeded in 6-well culture plates at a density of 5 × 10^5^ cells/ml. After incubation for 5 d until monolayer formation, heat-killed LAB strains at 1 × 10^9^ CFU/ml or PBS (for control) were treated to the cells. After incubation for 24 h, the supernatant was gathered by centrifugation at 14,000 ×*g* for 10 min and filtered through a 0.45 μm syringe filter. The CM was stored at –80°C until use.

### Cytotoxicity Measurement

MTT assay was conducted to evaluate cytotoxicity of CM using the method by Choi *et al*. [26]. SH-SY5Y cells were incubated in 96-well culture plates (1 × 10^5^ cells/well) for 24 h. Then, LAB–CM was added. Following incubation for 24 h, the medium was withdrawn and 100 μl of 0.5 mg/ml MTT solution was added. Later 4 h, the MTT solution was removed and DMSO was added to each well. Absorbance was evaluated at 570 nm, and cell viability was determined using Eq. (2).

$${\text{Cell viability (\%) =A}}_{\text{sample}}{\text{/A}}_{\text{control}}\text{×100}$$ (2)

where A_sample_ and A_control_ represent the absorbance of the cells treated with LAB–CM and the control treated with control–CM, respectively.

H_2_O_2_ (Sigma-Aldrich, USA) was used to induce cytotoxicity on SH-SY5Y cells. SH-SY5Y cells were seeded into 96-well culture plates at 1 × 10^5^ cells/well. After incubation for 24 h, the cells were treated with CM for 4 h and then exposed to H_2_O_2_ (150 μM) for 20 h. Then, the medium was removed and the cells were treated with MTT solution for 4 h. After removing the solution, DMSO was added to each well. Absorbance was evaluated at 570 nm, and cell viability was calculated using Eq. (2).

Protective effects were also confirmed by morphological observations. SH-SY5Y cells were plated at 1 × 10^5^ cells/well in 96-well culture plates. After treatment with CM and H_2_O_2_, the images of the cells were examined using a Nikon Eclipse Ti2-U fluorescence microscope (Nikon Co., Ltd., Japan) and a DS-Ri2 digital camera (Nikon Co., Ltd.).

### Assessment of Relative Gene Expression Using RT-PCR

Relative gene expression was measured by Choi *et al*. [26]. To measure the expression of *BDNF* in intestinal cells, HT-29 cells were inoculated in 6-well culture plates at a density of 1 × 10^6^ cells/well and incubated for 5 d. The HT-29 cells were incubated with heat-killed LAB (1 × 10^8^ CFU/well) for 24 h.

To detect the expression of *Bax*, *Bcl-2*, *BDNF*, and *TH* in neuroblastoma cells, SH-SY5Y cells were inoculated in 6-well culture plates at 1 × 10^6^ cells/well and incubated for 24 h. The SH-SY5Y cells pretreated with CM for 4 h, followed by treatment with 150 μM H_2_O_2_ for 3 h.

Total RNA was extracted using an RNeasy Mini Kit (Qiagen, Germany). cDNA was synthesized from isolated RNA using a cDNA synthesis kit (Thermo Fisher Scientific, USA). Gene expression was detected using SYBR Green PCR Master mix (Thermo Fisher Scientific) with real-time PCR (QuantStudio 1 Real-Time PCR, Thermo Fisher Scientific). RT-PCR was conducted as follows: initial denaturation at 95°C for 10 min, followed by annealing and extension as 40 cycles at 95°C for 20 s, 60°C for 20 s, and 72°C for 30 s. GAPDH (glyceraldehyde 3-phosphate dehydrogenase) was used as a reference gene. The results were analyzed using the 2^-ΔΔCt^ method. The PCR primers are listed in Table 1.

### Caspase-3 Activity Assay

The activity of caspase-3 was assessed using a caspase-3/cpp32 colorimetric assay kit (BioVision, USA). SH-SY5Y cells were seeded in 6-well culture plates at a density of 1 × 10^6^ cells/well. After 24 h of incubation, the cells were pretreated with CM for 4 h and then with H_2_O_2_ for 6 h. Following treatment, the cells were harvested and lysed with a cell lysis buffer. The cell lysates were centrifugated at 10,000 ×*g* for 1 min at 4°C. Then, 50 μl of cell lysis buffer containing 50 μg of protein was incubated with the same amount of 2 × reaction buffer comprising 10 mM DTT and 5 μl of 4 mM DEVD-*p*NA substrate at 37°C for 2 h. Absorbance was measured at 405 nm, and caspase-3 activity was decided by comparison with the control group.

### Statistical Analysis

All experiments were repeated in triplicate and are presented as the mean ± standard deviation. One-way analysis of variance and Duncan’s multiple-range test were used to compare multiple groups. The results were considered statistically significant at *p* < 0.05, and all statistical analyses were conducted using SPSS software (IBM, USA).

## Results

### Antioxidant Activities of Heat-Killed LAB Strains

Table 2 shows the antioxidant activities of the LAB strains. *L. brevis* KU15152 demonstrated the highest DPPH radical scavenging activity (14.25%) among the four heat-killed LAB strains. LGG indicated an activity close to that of *L. brevis* KU15152 (12.60%). However, those of *L. brevis* KU15159 and *L. brevis* KU15176 were 9.29% and 7.66%, respectively.

*L. brevis* KU15152 (52.85%) and *L. brevis* KU15159 (52.68%) showed the highest ABTS radical scavenging activities. LGG (50.42%) and *L. brevis* KU15176 (46.20%) showed lower values. Song *et al*. [28] demonstrated that the ABTS radical scavenging activities of heat-killed LGG and *Lactobacillus brevis* KCCM 12203P were 37.10%and 22.07%, respectively. Heat-killed *Lactobacillus brevis* B13-2 showed 47.43% ABTS radical-scavenging activity [23]. It is presumed that strains with antioxidant capabilities could have neuroprotective effects.

### Effect of Heat-Killed LAB Strains on H_2_O_2_-Stimulated Stress in SH-SY5Y Cells

**Fig. 1:**
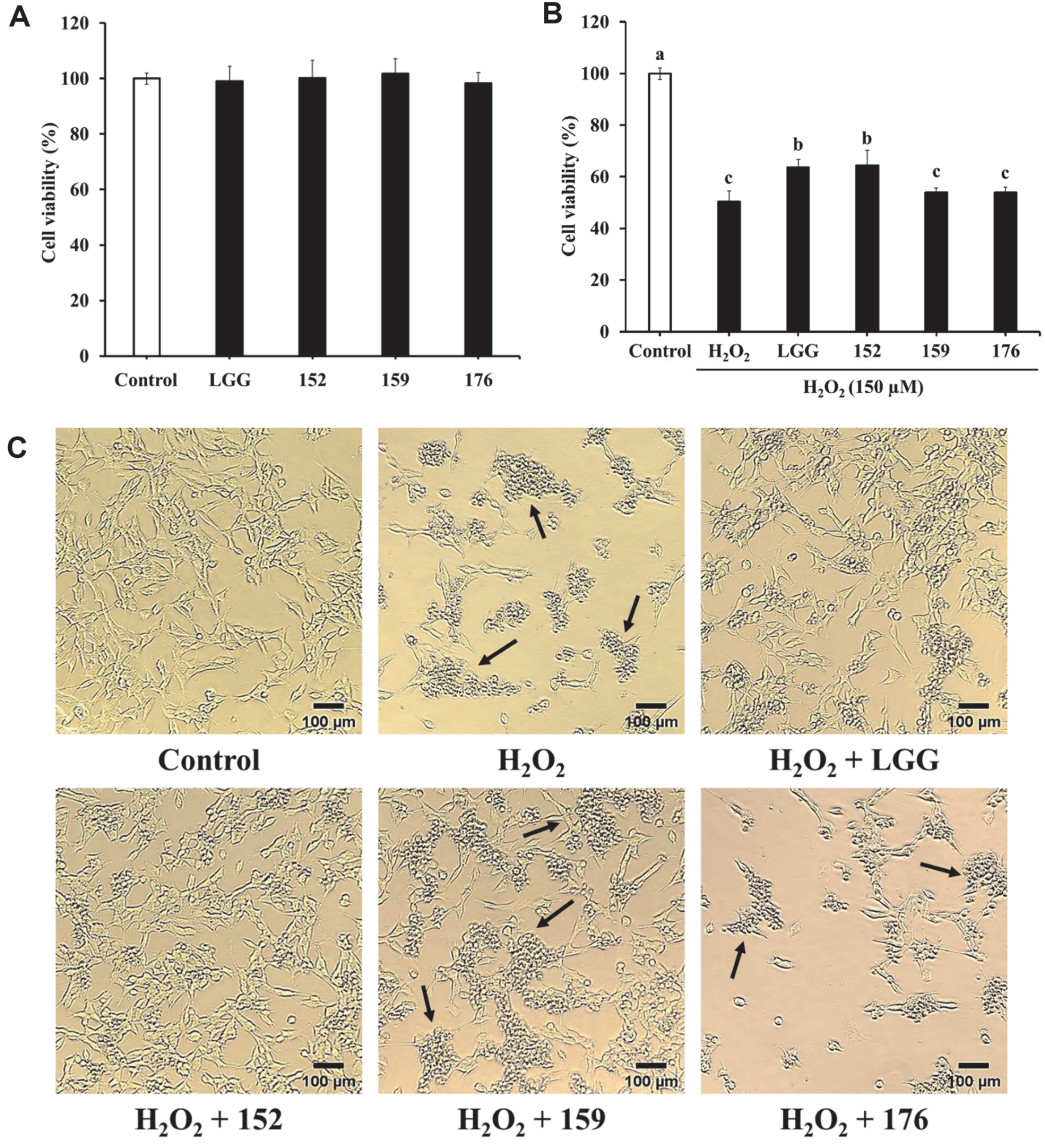
Neuroprotective effects of heat-killed LAB–CM on H_2_O_2_-induced toxicity in SH-SY5Y cells. (**A**) Effect of LAB–CM on cell viability in SH-SY5Y cells. (**B**) Effect of LAB–CM on cell viability of H_2_O_2_-treated SH-SY5Y cells. (**C**) Morphological changes in SH-SY5Y cells investigated using microscopy (magnification: 40×). Arrows indicate aggregation and shrinkage of SH-SY5Y cells. LGG, CM of heat-killed *L. rhamnosus* GG; 152, CM of heat-killed *L. brevis* KU15152; 159, CM of heat-killed *L. brevis* KU15159; 176, CM of heat-killed *L. brevis* KU15176. Data are presented as mean ± standard deviation of triplicate experiments. Different letters on the error bars represent significant differences (*p* < 0.05).

The cytotoxic effects of CM from heat-killed bacteria were assessed using an MTT assay. CM was found to be non-toxic to SH-SY5Y cells (Fig. 1A). As illustrated in Fig. 1B, the cell viability of samples treated with H_2_O_2_ only was 50.26%. In contrast, *L. brevis* KU15152–CM elevated the cell viability by 64.45%. The protective effect of *L. brevis* KU15152–CM was similar to that of heat-killed LGG (63.64%). *L. brevis* KU15159–CM (53.90%) and *L. brevis* KU15176–CM (53.93%) showed similar cell viability as H_2_O_2_-treated cells. Lee *et al*. [29] demonstrated that the CM of *Leuconostoc mesenteroides* H40 attenuated cell death against H_2_O_2_ in SH-SY5Y cells via the MTT assay. These results show that *L. brevis* KU15152 applies a neuroprotective effect against H_2_O_2_-induced toxicity.

Apoptotic cells exhibit morphological features, such as degradation of chromosomal DNA, shrinkage of cells, and DNA condensation [30]. The protective effects of CM were also compared using morphological observations (Fig. 1C). H_2_O_2_-treated SH-SY5Y cells showed aggregation and shrinkage of cell bodies. In contrast, pretreatment with heat-killed *L. brevis* KU15152–CM remarkably attenuated the cell damage. These observations implied that CM from heat-killed *L. brevis* KU15152 protected SH-SY5Y cells from H_2_O_2_-mediated toxicity.

### Effect of Heat-Killed LAB on mRNA Expression in HT-29 Cells

**Fig. 2:**
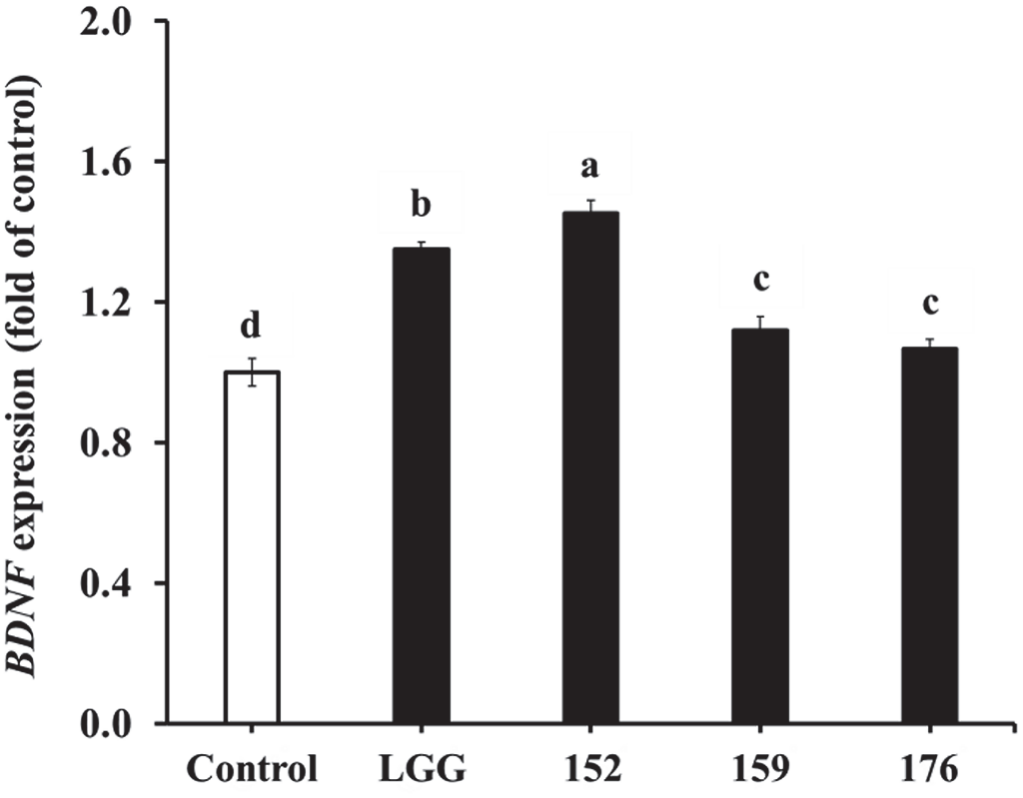
Effects of heat-killed LAB on *BDNF* expression in HT-29 cells, elucidated using RT-PCR. LGG, heatkilled *L. rhamnosus* GG; 152, heat-killed *L. brevis* KU15152; 159, heat-killed *L. brevis* KU15159; 176, heat-killed *L. brevis* KU15176. Data are presented as mean ± standard deviation of triplicate experiments. Different letters on the error bars represent significant differences (*p* < 0.05).

Cells treated with heat-killed LGG and *L. brevis* KU15152 indicated increased *BDNF* expression in HT-29 cells by 1.35- and 1.45-fold, respectively (Fig. 2). However, *L. brevis* KU15159 and *L. brevis* KU15176 showed relatively low *BDNF* expression, by 1.12- and 1.07-fold, respectively. *Lactobacillus buchneri* KU200793 increases *BDNF* expression in HT-29 cells [25]. *L. brevis* KU15152 was, thus, expected to have neuroprotective effects.

### Effect of Heat-Killed LAB–CM on mRNA Expression in SH-SY5Y Cells

**Fig. 3:**
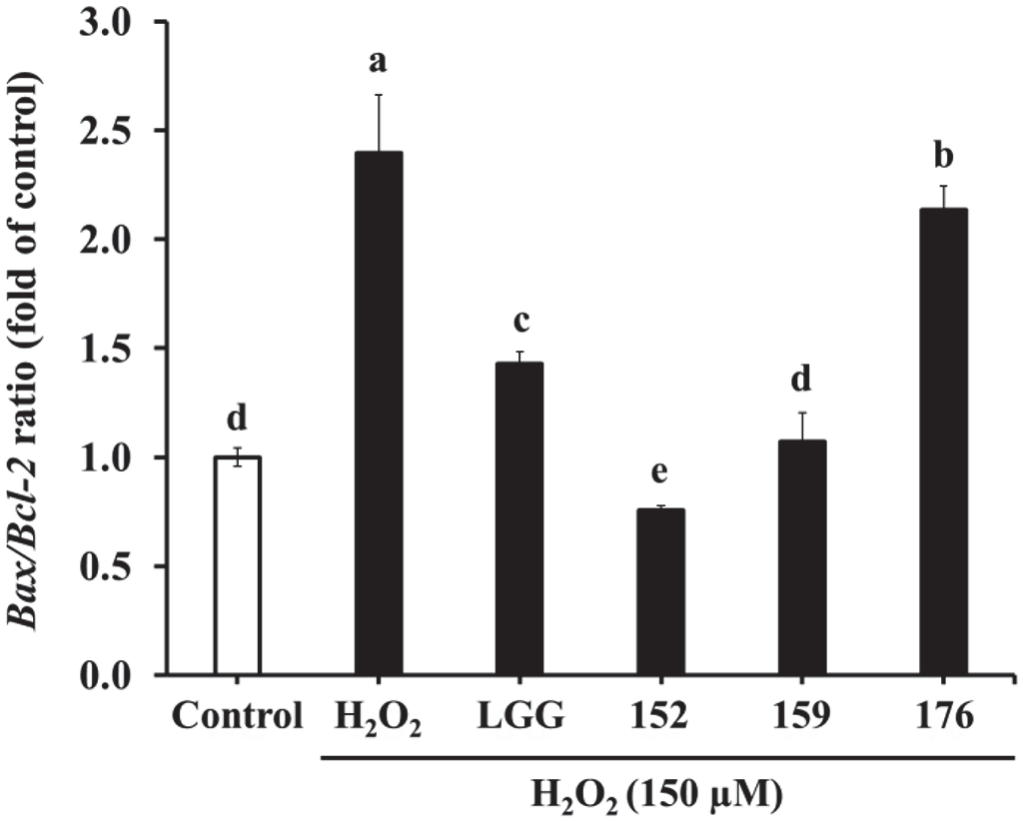
Effects of heat-killed LAB–CM on *Bax/Bcl-2* ratio related to apoptosis in SH-SY5Y cells by RT-PCR. LGG, CM of heat-killed *L. rhamnosus* GG; 152, CM of heat-killed *L. brevis* KU15152; 159, CM of heat-killed *L. brevis* KU15159; 176, CM of heat-killed *L. brevis* KU15176. Data are presented as mean ± standard deviation of triplicate experiments. Different letters on the error bars represent significant differences (*p* < 0.05).

As illustrated in Fig. 3, the *Bax/Bcl-2* ratio was considerably increased in the H_2_O_2_-treated cells by 2.40-fold compared to that in the H_2_O_2_-untreated cells. However, *L. brevis* KU15152–CM notably lowered this ratio by 0.76-fold. LGG, *L. brevis* KU15159, and *L. brevis* KU15176 showed 1.43-, 1.07-, and 2.14-fold increases, respectively. *Ruminococcus albus* CM decreases the *Bax/Bcl-2* ratio in H_2_O_2_-induced SH-SY5Y cells [31]. Thus, *L. brevis* KU15152 may alleviate mRNA expression related to apoptosis.

**Fig. 4:**
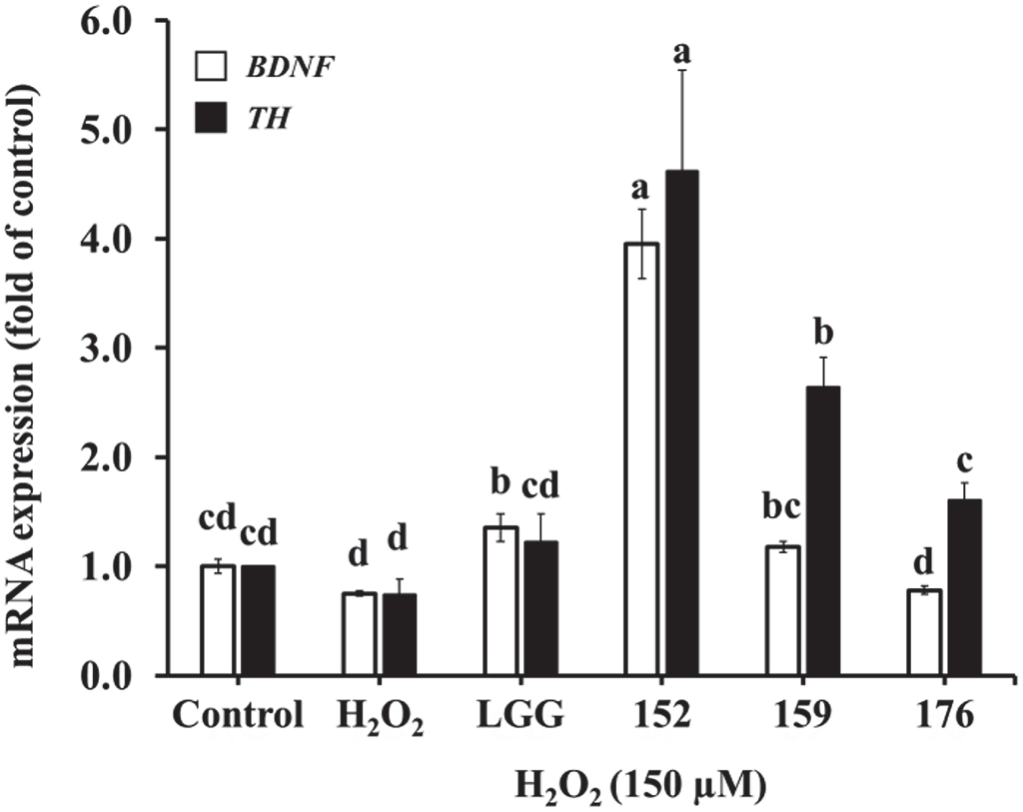
Effects of heat-killed LAB–CM on *BDNF* and *TH* expression in SH-SY5Y cells using RT-PCR. LGG, CM of heat-killed *L. rhamnosus* GG; 152, CM of heat-killed *L. brevis* KU15152; 159, CM of heat-killed *L. brevis* KU15159; 176, CM of heat-killed *L. brevis* KU15176. Data are presented as mean ± standard deviation of triplicate experiments. Different letters on the error bars represent significant differences (*p* < 0.05).

Fig. 4 shows the upregulation of *BDNF* and *TH* expression after treatment with CM. Cells treated with only H_2_O_2_ showed downregulated expression of *BDNF* and *TH* by 0.75- and 0.74-fold, respectively. *L. brevis* KU15152–CM dramatically increased *BDNF* and *TH* expression by 3.96- and 4.62-fold, respectively. The CM of LGG, *L. brevis* KU15159, and *L. brevis* KU15176 showed only 1.36-, 1.18-, and 0.78-fold *BDNF* expression, respectively. In addition, the three strains presented *TH* expression by 1.22-, 2.64-, and 1.61-fold, respectively. Jang *et al*. [32] suggested that the *Bifidobacterium adolescentis* NK98 and *Lactobacillus reuteri* NK33 enhanced hippocampal *BDNF* expression in a mouse model. In addition, *L. plantarum* PS128 ingestion demonstrated neuroprotective effects on dopaminergic neurons in a mouse model by restoring the expression of TH under MPTP (1-methyl-4-phenyl-1,2,3,6-tetrahydropyridine) toxicity [33]. Therefore, *L. brevis* KU15152 protects SH-SY5Y cells against oxidative stress by enhancing the expression of *BDNF* and *TH*.

### Effect of Heat-Killed LAB–CM on Caspase-3 Activity

**Fig. 5:**
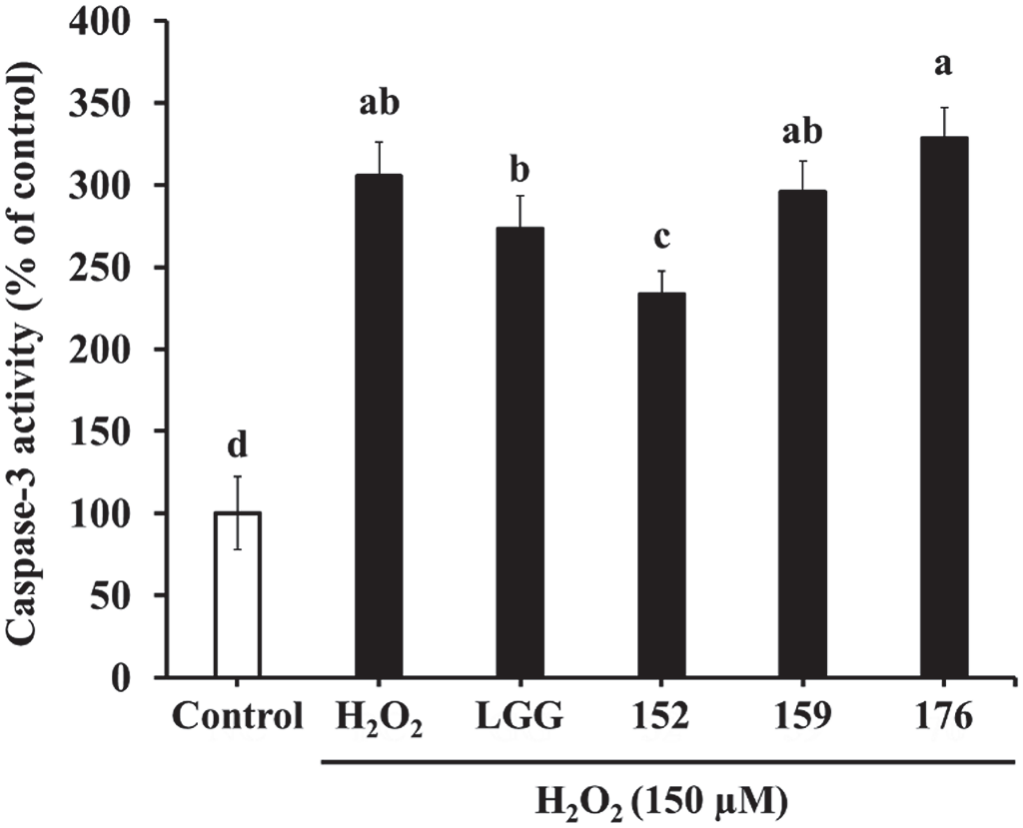
Effects of heat-killed LAB-CM on caspase-3 activity in the SH-SY5Y cells treated with H_2_O_2_ for 6 h. LGG, CM of heat-killed *L. rhamnosus* GG; 152, CM of heat-killed *L. brevis* KU15152; 159, CM of heat-killed *L. brevis* KU15159; 176, CM of heat-killed *L. brevis* KU15176. Data are presented as mean ± standard deviation of triplicate experiments. Different letters on the error bars represent significant differences (*p* < 0.05).

Fig. 5 shows the effects of caspase-3 after treatment with CM in SH-SY5Y cells. Treatment with H_2_O_2_ alone showed caspase-3 activity by 305.53% compared to that in untreated cells. However, the CM of *L. brevis* KU15152 significantly attenuated the enzyme activity by 233.33%. In contrast, LGG, *L. brevis* KU15159, and *L. brevis* KU15176 showed an increase in caspase-3 activity of 273.11, 295.57, and 328.26%, respectively. *L. plantarum* DP189 decreased caspase-3 activity in the substantia nigra of MPTP-induced PD mice [34]. These results confirm that *L. brevis* KU15152 inhibits H_2_O_2_-induced apoptosis in SH-SY5Y cells.

## Discussion

In this study, *L. brevis* KU15152 was used as heat-inactivated form and showed antioxidant capabilities. To measure the neuroprotective effects of heat-killed probiotic strain, the CM was used according to the GBA. The CM of heat-killed *L. brevis* KU15152 possibly protects SH-SY5Y cells against cytotoxicity. Additionally, treatment with *L. brevis* KU15152–CM upregulated the expression of neuroprotection-related genes and downregulated the expression of apoptosis-related genes. The CM of heat-killed *L. brevis* KU15152 also suppressed apoptosis-related enzyme activity. After the heat-inactivation of *L. brevis* KU15152, the various microbiological components such as exopolysaccharides, peptidoglycans, lipoteichoic acid, and metabolites were composited. It is assumed that the strain showed the neuroprotective effects because of the components [17].

Antioxidant properties are considered to contribute positively to neuroprotective effects [35]. The DPPH assay is subject to reducing the DPPH radical (purple color) to 1,1-diphenyl-2-picryl hydrazine (yellow color), and the ABTS assay is based on reducing the blue/green ABTS radical by antioxidants [36]. LGG, used as a reference strain, possesses strong antioxidant capacity [37]. In DPPH and ABTS assays, heat-killed *L. brevis* KU15152 showed antioxidant properties comparable to those of heat-killed LGG. These antioxidant activities were expected to contribute to the neuroprotective effects of heat-killed *L. brevis* KU15152.

Apoptosis, characterized by DNA fragmentation, membrane blebbing, nuclear condensation, and cell shrinkage, is a process of cell death [38]. H_2_O_2_ triggers apoptosis in neuronal cells and has generally been used to induce intracellular ROS generation and cell death [39]. It is expected that the neuroprotective effect of the heat-killed probiotic strain was attributed to the cell wall components including EPS as neurotransmitters [17]. The protective effects of LAB were assessed using MTT assay and morphological observations. Pretreatment with *L. brevis* KU15152–CM increased the suppressed cell viability that was induced by treatment with H_2_O_2_. The reduction in cell damage was confirmed by morphological observations.

Bcl-2, an anti-apoptotic Bcl-2 family member, exists in the outer mitochondrial membrane and regulates the release of cytochrome C [40]. Bax (pro-apoptotic factor), exist in the cytosol, which promotes permeabilization of the mitochondrial membrane, and accelerates apoptotic cell death [41]. The *Bax/Bcl-2* ratio is allowed to a better indicator of apoptosis than measuring the levels of Bax or Bcl-2, respectively. This ratio indicates the balance between anti- and pro-apoptotic proteins of the Bcl-2 family [42]. Treatment with H_2_O_2_ alone upregulated the *Bax/Bcl-2* ratio, whereas pretreated *L. brevis* KU15152–CM downregulated the ratio in SH-SY5Y cells. Therefore, *L. brevis* KU15152 can protect neuronal cells by controlling the expression of apoptosis-related genes.

BDNF is a crucial neurotrophic factor for the neurogenesis process, and its decreased expression has been considered a damaged motor ability in patients with PD [38]. BDNF plays a significant role in hippocampal long-term potentiation, memory formation, and plasticity [6]. TH is a rate-limiting enzyme in dopamine biosynthesis [38]. TH, which catalyzes the hydroxylation of tyrosine towards L-DOPA, has been considered a molecular agent for determining the level of dopamine [7]. In this study, *BDNF* expression was confirmed using RT-PCR in HT-29 cells. Among the four heat-killed LAB, *L. brevis* KU15152-treated cells exhibited the highest *BDNF* expression in HT-29 cells. According to the GBA, the strain increasing *BDNF* expression in intestinal cells could have neuroprotective effect. Moreover, the downregulated expression of *BDNF* and *TH* was markedly ameliorated by pretreatment with *L. brevis* KU15152–CM in the H_2_O_2_-treated SH-SY5Y cells.

Caspases regulate intracellular apoptotic signals followed by cellular oxidative stress [43]. Apoptosis induced by H_2_O_2_ includes caspase-3 activation, which occurs via the apoptotic caspase pathway [44]. H_2_O_2_-induced cell damage causes release of mitochondrial cytochrome C. The cytochrome C stimulates caspase-9 and leads to the activation of caspase-3, which in turn occurs DNA damage and cell death [45]. In the present study, caspase-3 activity was amplified after the treatment of H_2_O_2_ in SH-SY5Y cells and heat-killed *L. brevis* KU15152–CM suppressed caspase-3 activity. Despite the treatment of H_2_O_2_, *L. brevis* KU15152–CM decreased the caspase-3 activity by 72.20%. Thus, it can be considered that heat-killed *L. brevis* KU15152 effectively attenuate apoptosis induced by oxidative stress.

*L. brevis* KU15152 was isolated from a fermented Korean food. Heat-killed *L. brevis* KU15152 exhibited antioxidant activity similar to that of LGG. CM prepared from heat-killed *L. brevis* KU15152 increased cell viability and alleviated morphological damage in SH-SY5Y cells. In addition, heat-killed *L. brevis* KU15152 demonstrated high *BDNF* expression in HT-29 cells, and the highest expression of *BDNF* and *TH* was observed after treatment with *L. brevis* KU15152–CM in SH-SY5Y cells. Heat-killed *L. brevis* KU15152–CM also reduced the expression of apoptosis-related genes and caspase-3 activity. These neuroprotective effects were expected to attribute to the cell components from parabiotic *L. brevis* KU15152 according to the GBA. Consequently, heat-killed *L. brevis* KU15152 could be used as food ingredients to prevent neurodegenerative diseases. However, in vivo experiments are required for further study.

## Figures and Tables

**Table 1:** Primer sequences used in RT-PCR.

Primer^1^		Primer sequence (5’-3’)	Reference
*BDNF*	Sense	ATGACCATCCTTTTCCTTACT	[27]
	Antisenese	GCCACCTTGTCCTCGGAT	
*TH*	Sense	GAGGAGAAGGAGGGGAAG	[27]
	Antisenese	ACTCAAACACCTTCACAGCT	
*Bax*	Sense	GTGGTTGCCCTCTTCTACTTTGC	[25]
	Antisenese	GAGGACTCCAGCCACAAAGATG	
*Bcl-2*	Sense	CGGCTGAAGTCTCCATTAGC	[25]
	Antisenese	CCAGGGAAGTTCTGGTGTGT	
*GAPDH*	Sense	GAGTCAACGGATTTGGTCGT	[25]
	Antisenese	GACAAGCTTCCCGTTCTCAG	

^1^*BDNF*, brain-derived neurotrophic factor; *TH*, tyrosine hydroxylase; *Bax*, Bcl-2 associated X protein; *Bcl-2*, B-cell lymphoma-2; *GAPDH*, glyceraldehyde 3-phosphate dehydrogenase.

**Table 2:** Antioxidant activities of the Lactobacillus strains.

Antioxidant activity	Heat-killed Lactobacillus strains			
	LGG^1^	*L. brevis* KU15152	*L. brevis* KU15159	*L. brevis* KU15176
DPPH radical scavenging activity (%)	12.60 ± 1.61^a^	14.25 ± 1.86^a^	9.29 ± 1.24^b^	7.66 ± 2.49^b^
ABTS radical scavenging activity (%)	50.42 ± 1.54^b^	52.85 ± 3.15^a^	52.68 ± 1.27^a^	46.20 ± 1.42^c^

^1^LGG, *L. rhamnosus* GG. ^a–c^Different letters in the same row indicate significant differences (*p* < 0.05).
